# Comprehensive Approach to Improving Maternal Health and Achieving MDG 5: Report from the Mountains of Lesotho

**DOI:** 10.1371/journal.pone.0042700

**Published:** 2012-08-27

**Authors:** Hind Satti, Sophie Motsamai, Palesa Chetane, Leshoboro Marumo, Donna J. Barry, Jennifer Riley, Megan M. McLaughlin, Kwonjune J. Seung, Joia S. Mukherjee

**Affiliations:** 1 Partners In Health Lesotho, Maseru, Lesotho; 2 Department of Global Health and Social Medicine, Harvard Medical School, Boston, Massachusetts, United States of America; 3 Partners In Health, Boston, Massachusetts, United States of America; 4 Division of Global Health Equity, Brigham and Women's Hospital, Boston, Massachusetts, United States of America; Vanderbilt University, United States of America

## Abstract

**Background:**

Although it is now widely recognized that reductions in maternal mortality and improvements in women's health cannot be achieved through simple, vertical strategies, few programs have provided successful models for how to integrate services into a comprehensive program for maternal health. We report our experience in rural Lesotho, where Partners In Health (PIH) in partnership with the Ministry of Health and Social Welfare implemented a program that provides comprehensive care of pregnant women from the community to the clinic level.

**Methods:**

Between May and July 2009, PIH trained 100 women, many of whom were former traditional birth attendants, to serve as clinic-affiliated maternal health workers. They received performance-based incentives for accompanying pregnant women during antenatal care (ANC) visits and facility-based delivery. A nurse-midwife provided ANC and delivery care and supervised the maternal health workers. To overcome geographic barriers to delivering at the clinic, women who lived far from the clinic stayed at a maternal lying-in house prior to their expected delivery dates. We analyzed data routinely collected from delivery and ANC registers to compare service utilization before and after implementation of the program.

**Results:**

After the establishment of the program, the average number first ANC visits increased from 20 to 31 per month. The clinic recorded 178 deliveries in the first year of the program and 216 in the second year, compared to 46 in the year preceding the program. During the first two years of the program, 49 women with complications were successfully transported to the district hospital, and no maternal deaths occurred among the women served by the program.

**Conclusions:**

Our results demonstrate that it is possible to achieve dramatic improvements in the utilization of maternal health services and facility-based delivery by strengthening human resource capacity, implementing active follow-up in the community, and de-incentivizing home births.

## Introduction

Lesotho is making commendable progress toward a number of the Millennium Development Goals (MDG). However, in the country and elsewhere in the developing world, there has been a marked lack of progress toward MDG 5, to improve maternal health. One of the two targets of MDG 5 is to achieve a 75% reduction in the maternal mortality ratio (MMR)—death associated with pregnancy or child birth—from 1990 to 2015. As of 2008, however, the MMR in Lesotho is among the highest in the world and increased from 237 to 964 maternal deaths per 100,000 live births between 1990 and 2008, respectively [1].

At the 2010 MDG summit held at the United Nations, it was recognized that among the seven Millennium Development Goals, the least progress has been made toward achieving MDG 5 [2]. The emerging consensus is that improvement in women's health cannot be made through simple, vertical strategies; rather, it requires broad-based health system strengthening at every level of care, from the community to the clinic to the hospital [3]. Comprehensive programs are needed that incorporate family planning at all levels of care, that link the availability of emergency obstetrical services to ambulatory maternity care, and that integrate maternity services with HIV, tuberculosis (TB), and malaria efforts [4], [5].

In this paper, we report our experience in rural Lesotho, where Partners In Health (PIH) in partnership with the Lesotho Ministry of Health and Social Welfare (MOHSW) has implemented a pilot program that provides comprehensive care of pregnant women from the community to the health center level, linking key primary care services (include HIV testing and treatment) to antenatal care (ANC) and facility-based delivery. We describe key challenges and present encouraging data on initial utilization of services from the first two years of the pilot program.

### Context

Lesotho is a small highland country completely surrounded by South Africa, with a population of approximately 1.8 million. Seventy-seven percent of the population lives in rural areas [6]. More than 60% of the country is mountainous, and many villages remain accessible only by foot or horseback, which presents a major challenge to promoting facility-based delivery for pregnant women. In addition to the challenges presented by the terrain, Lesotho has one of the highest burdens of HIV infection in the world, with an adult prevalence of 24%, and 58.9% of maternal deaths are estimated to be HIV-related [6], [7]. Moreover, the burden of TB is high, with an estimated prevalence of 402 cases per 100,000 population [8].

As a result of geographic and economic barriers, many women in rural Lesotho continue to lack adequate access to ANC and delivery services. In rural villages, traditional birth attendants generally are not integrated into the health system and therefore are not referring women for HIV testing, antenatal care, or other needed health services. Moreover, because traditional birth attendants are paid or compensated for assisting women delivering at home, they are often reluctant to refer women for facility-based delivery.

## Methods

In 2009, Elton John AIDS Foundation UK granted PIH Lesotho funding to implement and evaluate a program to improve access to HIV care and reproductive health services for women in rural Lesotho. The goal of the program was to increase utilization of antenatal services, facility-based delivery, and HIV case detection and treatment of pregnant women in the mountains of Lesotho. The program was first piloted in May 2009 in the catchment area of Bobete health center, a MOHSW clinic located in the mountains in Thaba-Tseka district that PIH has supported since 2007. Bobete health center serves 71 villages, with an estimated total catchment area of 25,000 people, including approximately 7,000 women of reproductive age. Results from the 2009 Demographic and Health Survey show that access to skilled birth attendants is lower in Thaba-Tseka district compared to nationwide: 57% women deliver at home (vs. 40% nationwide), and 43% are assisted by skilled birth attendants (vs. 61%) [6].

**Figure 1:**
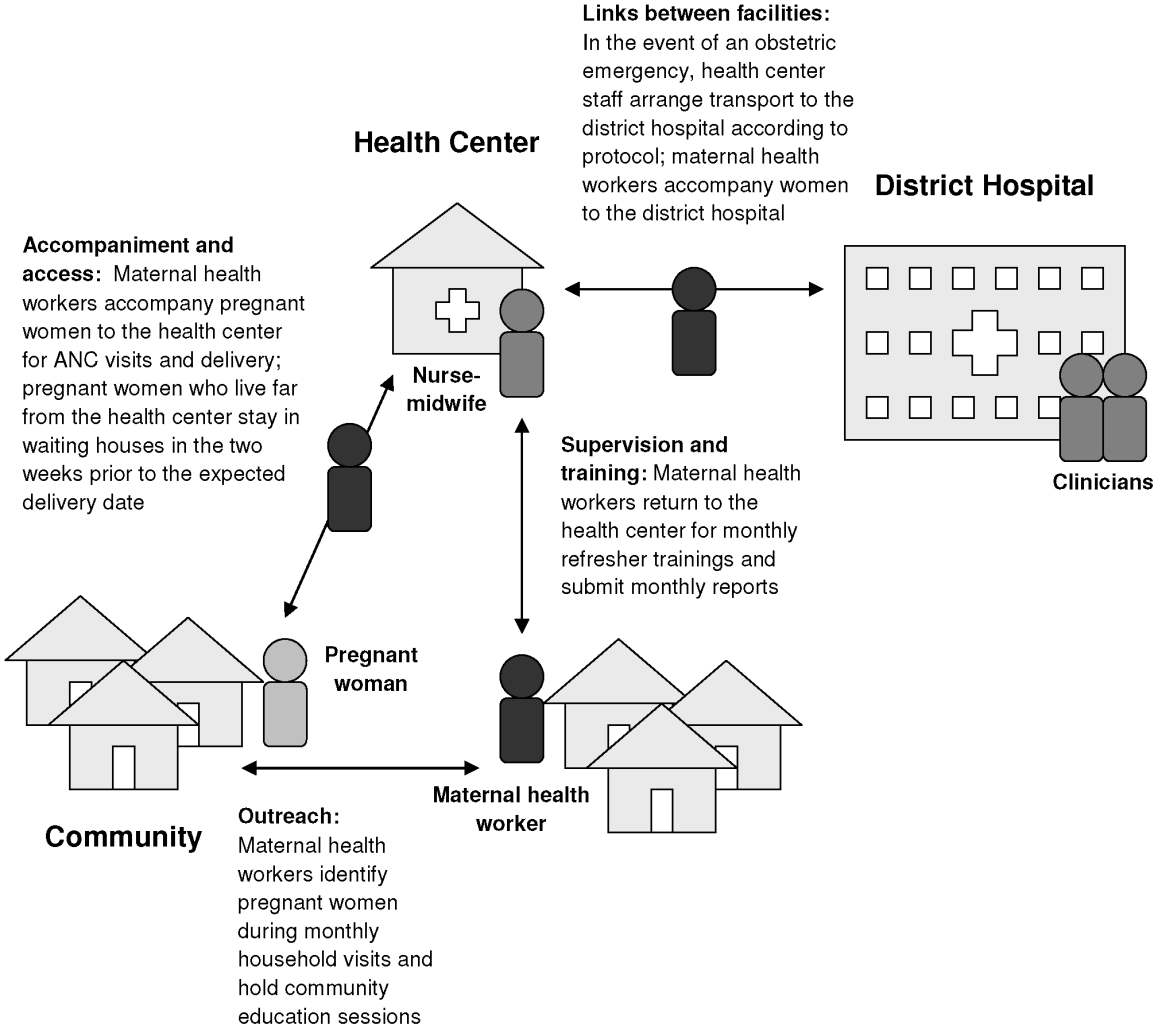
Schematic of the continuum of care for maternal health.

To implement the program both in the community and at the health center level, human resources were augmented and an incentive system was piloted to encourage ANC and facility-based delivery. At the community level, PIH trained 100 maternal health workers, between May and July 2009, to identify pregnant women and accompany them to the health center for ANC, delivery, and postpartum services (Figure 1). They also provide education about the program during community gatherings, emphasizing the importance of facility-based care for pregnant women and newborns.

The maternal health workers were selected in consultation with the village chiefs to ensure community acceptability, and selection criteria included basic literacy and the ability to walk long distances. Many of the women selected were formerly traditional birth attendants. Historically, the Lesotho MOHSW hired women to serve as traditional birth attendants, training them in safe delivery practices and providing them with some basic supplies to conduct home deliveries. The MOHSW has since abandoned this policy. In some communities, traditional birth attendants have acquired their skills through apprenticeship, generally from older women in their families, and have not received any previous formal training.

**Figure 2:**
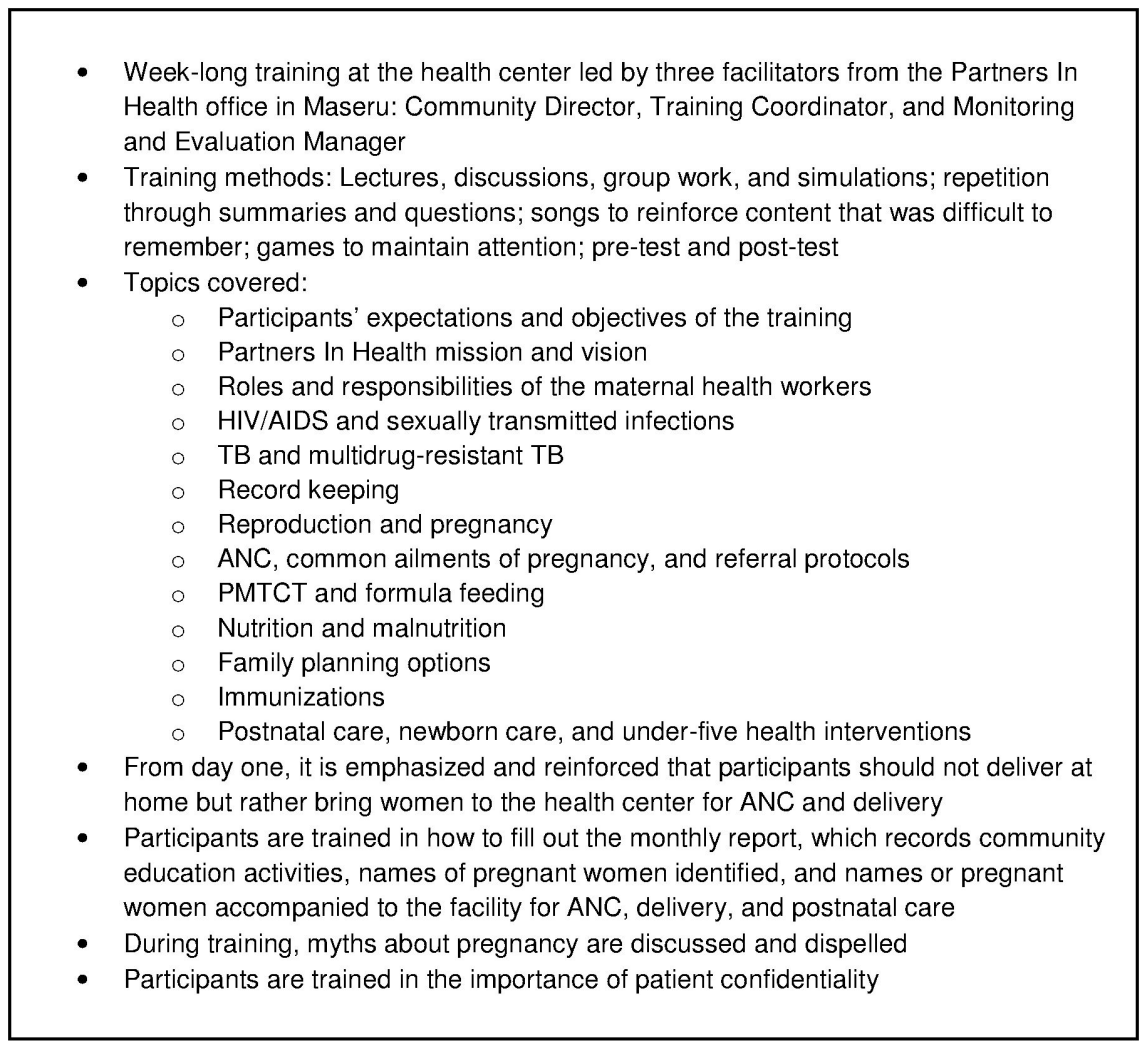
Curriculum for training maternal health workers.

The new maternal health workers received a seven-day training, covering specific topics of maternal health (Figure 2) in addition to the standard PIH community health worker curriculum, which has been extensively field tested in Haiti, Rwanda, Malawi, and Lesotho [9], [10]. The maternal health workers received exercise books for recording their activities, and the training included practice filling out these records. They continue to return to Bobete health center monthly for refresher training.

In addition, in their role as clinic-affiliated maternal health workers (as opposed to traditional birth attendants), the maternal health workers received performance-based incentives to promote the longitudinal accompaniment of women—i.e. attendance at antenatal clinic, facility-based delivery, and a postpartum follow-up visit. All maternal health workers are required to maintain a monthly record of their activities, including the number of patients counseled about attending the health center for ANC and delivery and the number of patients physically accompanied to the health center for these services. The supervising nurse-midwife also maintains records of the dates that the maternal health workers accompanied women to the health center, which are used to verify the maternal health workers' records.

The maternal health workers receive a performance-based salary on a monthly basis: 100 rand (US$12) for attending monthly trainings and submitting monthly reports, 100 rand (US$12) for accompanying women for a first ANC visit, 50 rand (US$6) for accompanying women for a subsequent ANC visit, and 200 rand (US$24) for accompanying women to the health center for delivery. The payment is comparable to the monthly salaries paid to community health workers employed by the Lesotho MOHSW (300 rand). As an additional incentive, mothers who attend all ANC visits, are tested for HIV, and deliver at the clinic receive “new baby starter packs” – a package of clothing and hygiene items to help care for the newborn.

At Bobete health center, previously, a nursing assistant was responsible for ANC, and few deliveries took place at the clinic each month. As part of the maternal mortality reduction program, a nurse-midwife was hired to provide ANC and delivery care at Bobete health center and to train and supervise the new maternal health workers.

**Figure 3:**
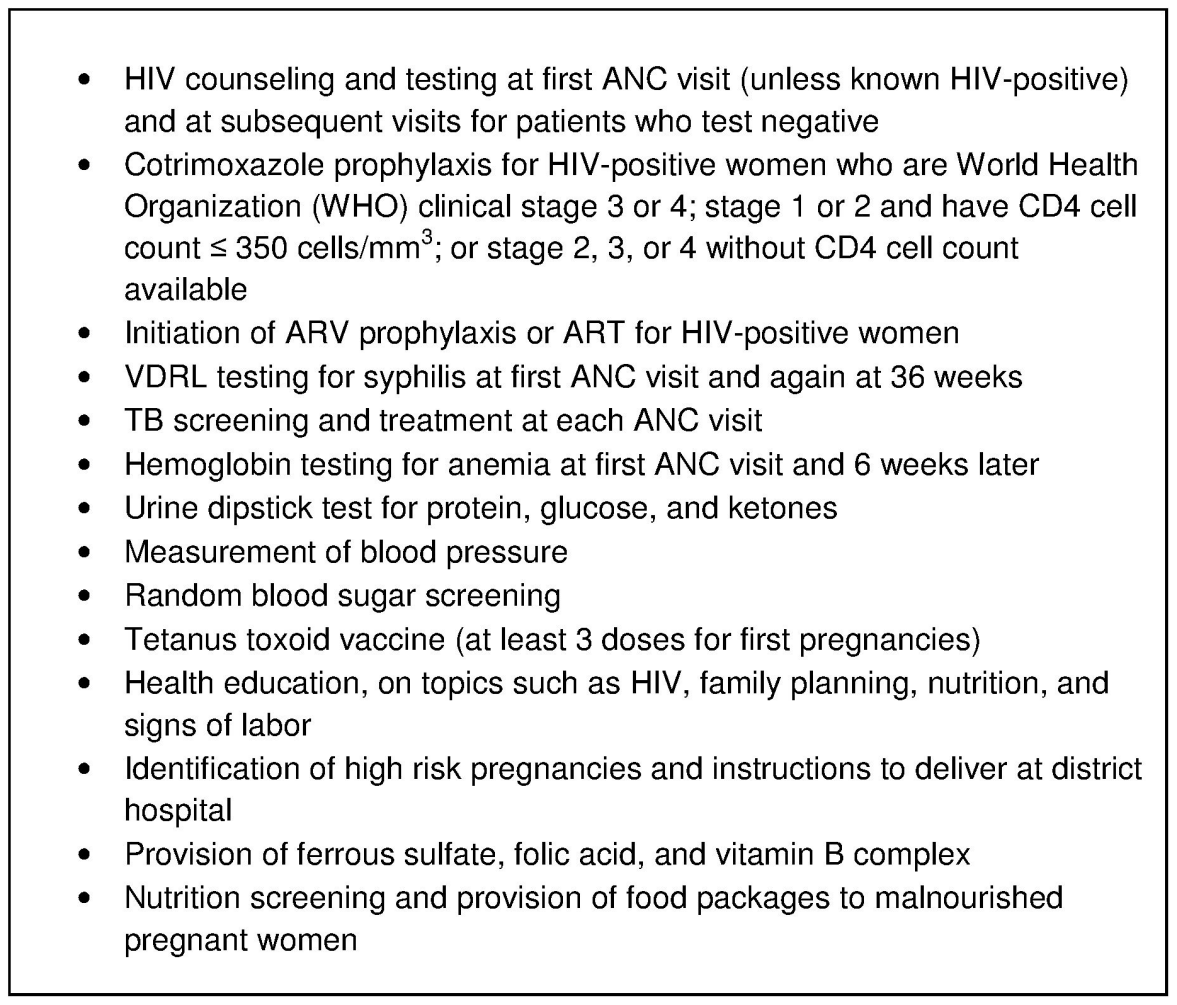
Components of antenatal care provided at the health center.

The ANC provided at Bobete is comprehensive, integrating risk screening, health education, tetanus immunization, family planning counseling, pap smears, and testing and treatment for TB, HIV, and other sexually transmitted infections delivered as a “one-stop shop” (Figure 3). The health center staff was trained in clear protocols for referring women with maternal complications and obstetric emergencies to the district hospital.

To overcome the geographic barriers to delivering at the clinic, PIH established a maternal waiting house (lying-in center) on the grounds of the clinic, which accommodates twelve women at a time. Women who live more than a two-hour walk from the clinic or face other geographic difficulties traveling to the clinic are invited to stay in the house in the two weeks prior to their expected delivery dates. The maternal health workers accompany women for admission to the waiting house, visit them regularly during their stay, and accompany them back to their villages after delivery. An on-site cook prepares three meals per day for the women staying in the house. A checklist of medications, supplies, and equipment for ANC, the delivery room, and maternal waiting houses ensures that the health center is adequately equipped to provide these services and ensure safe deliveries.

All women who test positive for HIV are offered antiretroviral therapy (ART) or antiretroviral prophylaxis and counseled on their infant feeding options, in accordance with Lesotho MOHSW guidelines [11]. Women who choose replacement feeding are provided with formula, a gel stove, fuel, bottles, thermos, soap, and cleaning brush to ensure sanitary formula feeding practices. The maternal health workers are trained in safe formula feeding and exclusive breastfeeding and work with mothers to ensure they know how to use these techniques. All infants born to HIV-positive mothers who are formula-fed are tested by DNA polymerase chain reaction (PCR) at six weeks and nine months after birth. Infants who are exclusively breastfed are also tested at six weeks after the end of breastfeeding.

Maternal health workers are also responsible for ensuring adequate postpartum care after delivery, which includes pap smears and family planning counseling and services. Women who deliver at the health center remain there for a 48-hour observation period. Women who are unable to get to the clinic in time and deliver at home are accompanied by the maternal health worker to the health center within 72 hours of delivery. HIV-exposed infants are accompanied to the health center for evaluation one week after delivery, and all women are accompanied for a postpartum visit six weeks after delivery.

A strong monitoring and evaluation component has been integral to the program since its inception. Before the program was established, community health workers carried out a village survey in order to collect baseline household data on women of reproductive age. As part of their duties, the maternal health workers now conduct active monthly surveillance in the village to which they are assigned. Every month they visit each household in the village, identifying women who may be pregnant and encouraging them to visit the health center for evaluation. The program is now capturing data on home deliveries and maternal deaths in the community, which previously went unrecorded.

The program is being implemented as part of a broader continuum of health services. Community health workers will continue to follow the mother and child for five years after the delivery, ensuring access to family planning and child health services, including childhood vaccines, monthly monitoring for malnutrition, and treatment of malnutrition.

### Ethics Statement

We analyzed data routinely collected from delivery and ANC registers for monitoring and evaluation purposes to compare service utilization before and after implementation of the program. This study was approved by the Partners HealthCare Human Research Committee. In the approved protocol, the requirement for informed consent was waived, since this was a retrospective study of information previously collected in the course of routine clinical care.

## Results

The number of women attending ANC services at Bobete health center increased significantly since the program was established. In the year preceding the program, from June 2008 to May 2009, Bobete health center recorded a monthly average of 20 first ANC visits (of the woman's current pregnancy). In the first year of the program, the average number of first ANC visits increased to 32 per month, and in the second year, Bobete recorded an average of 31 first ANC visits per month.

The increase in ANC coverage allowed health center staff to identify HIV-infected women who might otherwise have gone undiagnosed and to provide them with services for the prevention of mother-to-child transmission (PMTCT) of HIV. In the two years of the program, HIV testing and counseling at the first ANC visits reached over 94% of women whose HIV status was unknown at the time of their first ANC visit (Table 1). In the year prior to the program, Bobete health center recorded 18 new PMTCT clients who were registered at ANC. In the first two years of the program, 103 women initiated antiretroviral prophylaxis or ART at ANC, and 68 PMTCT clients delivered in a facility.

**Table 1:** Utilization of services in the first two years of the program, June 2009–May 2011.

Service		No. of women served	Coverage
First ANC visits		748	
	Hemoglobin testing	637	85% of first ANC visits
	VDRL (syphilis) testing	644	86% of first ANC visits
	HIV status known before ANC	194	26% of first ANC visits
	HIV testing at ANC	520	94% of women with unknown status at first ANC visit
	HIV serostatus positive	256	34% of first ANC visits
Facility deliveries		394	
	Referrals to hospital for complications	49	12% of facility deliveries
	Admissions to maternal waiting houses	218	55% of facility deliveries

Hospital referral data unavailable for the year prior to the program (June 2008–May 2009).

**Figure 4:**
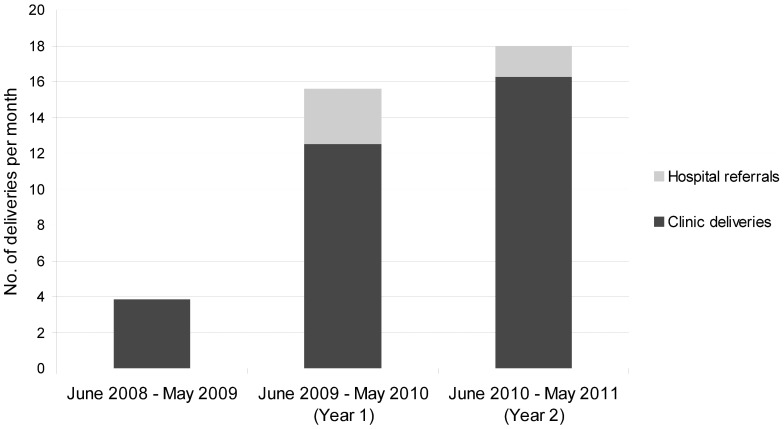
Average number of facility-based deliveries per month, by program year.

The number of women delivering at Bobete health center increased dramatically after the program was established. In the first year of the program, Bobete recorded 178 deliveries, including referrals to the district hospital, compared to 46 in the previous year. In the second year, Bobete recorded 216 deliveries. Figure 4 shows the increase in the average monthly number of facility deliveries by program year. Utilization of the maternal waiting houses has also increased, from 96 women during the first year to 122 women in year two.

During the two years of the program, 49 women who were at high risk of complications or experienced complications during delivery at the health center, such as premature rupture of the membranes or prolonged second stage labor, were successfully transported to the district hospital for obstetric care. During this time, no maternal deaths occurred among the women enrolled in the program, and the maternal health workers reported no maternal deaths during home births in the catchment area of Bobete health center.

## Discussion

Our results from the first two years of the program demonstrate that even in a geographically challenging setting, it is possible to achieve dramatic improvements in the utilization of maternal health services and facility-based delivery through a combination of strengthening human resource capacity, providing incentives for facility based delivery, re-purposing traditional birth attendants as community health workers with performance-based incentives and connection to the clinic, and improving infrastructure to support facility-based birth. Based on our experience, continuity of care between the community and health facilities, an integrated package of services, and expanded access to health care during all phases of women's reproductive years are effective for improving maternal health.

Our experience in Lesotho illustrates the benefits of integrating a variety of health services for women at the health center. In settings with high burdens of HIV and TB, testing and treatment for both diseases must be integrated into all phases of antenatal, intrapartum, and postpartum care to improve the long-term health of the mother and child.

Within a comprehensive approach, there must be a focus on the intrapartum period, where much of the most readily preventable mortality and morbidity occurs [5]. Timely referral and transport of women with complications to the district hospital, where emergency obstetric care was available, were key to achieving zero maternal deaths. Elements of the program addressed each of the three delays in the framework often used for describing the causes of maternal mortality: delay in making the decision to seek care when experiencing an obstetric emergency, delay in reaching an appropriate obstetric facility once the decision has been made to go, and delay in receiving adequate care once the facility has been reached [12], [13]. Screening during ANC ensured that the most at-risk women were identified early. Each visit incorporated counseling about the importance facility deliveries. Maternal waiting houses for women who live more than two hours from the facility or face other geographic difficulties traveling to the clinic were built and staffed, and clear protocols on referral and transport to the district hospital were established.

Our program also demonstrates that community health workers specifically responsible for women's health can play a key role in increasing uptake of maternal health services by linking the community and health facilities. While the strategy of working with traditional birth attendants is a subject of debate in the global health community [14], [15], we successfully re-purposed a cadre of traditional birth attendants, training them to be community health workers and paying them monthly for their work, as well as providing incentives for women completing ANC visits, facility-based delivery, and postpartum care. This financial package and engagement with the clinic serves to change the incentive from conducting home births to identifying pregnant women in the community and accompanying them to the clinic. By providing job security, the program not only sustained their livelihood but also motivated former traditional birth attendants to encourage facility deliveries rather than home births.

A number of home deliveries continue to occur in the catchment area each month, but overall the former traditional birth attendants have been cooperative in working with the nurse-midwife to increase facility-based care. Initially, however, the program was met with resistance from the traditional birth attendants. During community meetings prior to program implementation, they demanded supplies for conducting deliveries. PIH staff explained the dangers of home deliveries, emphasized the high MMR in Lesotho, and encouraged traditional birth attendants to share anecdotes about maternal deaths they had witnessed in their villages. These initial discussions, particularly the anecdotes, were effective in securing the cooperation of the traditional birth attendants. Training also proved to be challenging, because many of them had not received any previous formal training and lacked basic knowledge about HIV, TB, and malnutrition. In the first months of the program, the former traditional birth attendants often did not write their reports correctly, since they had never been required to report their activities previously, and the supervising nurse-midwife had to work closely with them to improve their reporting.

The number of women delivering at facilities increased more than threefold in the first year of the program and continues to increase. However, we have not yet met our estimated targets for monthly new ANC visits and facility deliveries based on the household survey results, and we continue to strive for universal coverage in the catchment area. Success breeds new challenges, and as the number of women reached increases, so does the need for expanded facilities and additional human resources. Moreover, bringing women into the formal health system is not sufficient; efforts at continually improving the quality of services are essential for continued program success. We have used lessons learned from the pilot program in Bobete to inform expansion of the program to other PIH-supported clinics in Lesotho.

The major limitation of this study is the reliance on facility-based data. Because of the lack of population-level data before and after implementation, we have not been able to quantify the impact of the program on maternal deaths, neonatal deaths, and unplanned pregnancies in the catchment area. As the program matures and expands to additional sites, we plan to report on additional indicators of the program's effectiveness, including newborn outcomes such as HIV-free survival among HIV-exposed infants and percentage of low birth weight infants, which has recently been added to the program's monthly reports. Qualitative studies examining how the families of pregnant women perceive the program and how maternal health workers who were formerly traditional birth attendants perceive their new roles are needed. We have also begun to take steps toward implementing a unique identifier for women and their children in order to analyze the association between receipt of maternal health services and newborn and child health outcomes. This system would also allow us to link data from ANC, delivery, and postnatal records in order to report measures of program retention, such as the percentage of women completing the cascade of maternal health services (four ANC visits, facility delivery, and postnatal visit) among those enrolled in the program.

To achieve MDG 5 in countries such as Lesotho, comprehensive programs are needed. In 1997, at the 10^th^ anniversary meeting of the Safe Motherhood Initiative, consensus emerged that a full spectrum of interventions, including skilled attendance at delivery, was needed in order to reduce maternal mortality [16]. More than a decade later, although evidence for individual interventions to reduce maternal mortality has grown, few programs have provided successful models for how to integrate these individual interventions into a comprehensive program for maternal health. We hope that presenting this model, including our ambitions for improved coverage and access, will be valuable to others attempting to address this pressing challenge.
